# Potential Therapeutic Effects of Exosomes Packed With a miR-21-Sponge Construct in a Rat Model of Glioblastoma

**DOI:** 10.3389/fonc.2019.00782

**Published:** 2019-08-20

**Authors:** Hamideh Monfared, Yavar Jahangard, Maryam Nikkhah, Javad Mirnajafi-Zadeh, Seyed Javad Mowla

**Affiliations:** ^1^Department of Molecular Genetics, Faculty of Biological Science, Tarbiat Modares University, Tehran, Iran; ^2^Department of Nanobiotechnology, Faculty of Biological Sciences, Tarbiat Modares University, Tehran, Iran; ^3^Department of Physiology, Faculty of Medical Science, Tarbiat Modares University, Tehran, Iran

**Keywords:** glioblastoma, microRNA, miR-21, exosomes, sponge

## Abstract

Glioblastoma multiforme (GBM) is a grade 4 and the most aggressive form of glioma, with a poor response to current treatments. The expression of microRNAs (miRNAs) is widely dysregulated in various cancers, including GBM. One of the overexpressed miRNAs in GBM is miR-21 which promotes proliferation, invasion and metastatic behaviors of tumor cells. With a size of 30–100 nm, the extracellular vesicles “exosomes” have emerged as a novel and powerful drug delivering systems. Recently, exosomal transfer of miRNAs or anti-miRNAs to tumor cells has introduced a new approach for therapeutic application of miRNAs to combat cancer. Here, we have tried to down-regulate miR-21 expression in glioma cell lines, U87-MG, and C6, by using engineered exosomes, packed with a miR-21-sponge construct. Our data revealed that the engineered exosomes have the potential to suppress miR-21 and consequently to upregulate miR-21 target genes, *PDCD4* and *RECK*. Interestingly, in cells treated with miR-21-sponge exosomes we observed a decline in proliferation and also an elevation in apoptotic rates. Finally, in a rat model of glioblastoma, administrating exosomes loaded with a miR-21-sponge construct leads to a significant reduction in the volume of the tumors. In brief, our findings suggest a new therapeutic strategy to use engineered exosomes to deliver a miR-21-sponge construct to GBM cells, in order to block its malignant behavior.

## Introduction

Glioblastoma multiforme (GBM) is the most aggressive form of brain tumors/gliomas. Glioma is divided into 4 grades and GBM is the grade 4, according to the World Health Organization (WHO) classifications (1). The life expectancy of the GBM patients is ~18 months after diagnosis (2). In GBM, similar to other cancer types, the communication between tumor cells and its microenvironment has a big impact on tumor progression; and hence it provided a new approach for cancer treatment (3, 4).

Extracellular vesicles (Evs) are membrane fragments shed from cell surfaces to transfer cytoplasmic or membrane contents to neighboring cells or body fluids. Evs contain numerous proteins, lipids, DNAs, mRNAs, and various kinds of non-coding RNAs (5–7). Exosomes are a subclass of EVs with a size of 30–100 nm, with a recognized role as an important mediator of signaling event between tumor cells and other cells in its microenvironment (8, 9). One of the exosomes' components with an important signaling role in cancer progression is microRNAs (miRNAs) (10–13).

miRNAs are small (~22 nt) non-coding RNAs involved in diverse physiological and developmental processes, and their dysregulation lead to several diseases including cancer (14–16). miR-21 is a well-known miRNA, found to be overexpressed in almost all cancer types, where its upregulation promotes cell proliferation, invasion, and metastatic behavior. miR-21 targets several genes such as *PDCD4, TIMP3*, and *RECK*, which are key regulators of apoptotic and metastatic pathways (17–23). Concerning its global oncogenic role, miR-21 has recently been proposed as a suitable target for GBM therapy. Inhibition of miR-21 via different strategies elevated apoptotic cell death, sensitivity to chemotherapy/radiotherapy, and diminished tumor progression (24–28).

It has already been shown that miRNA inhibition using decoy or sponge-like constructs have a potential therapeutic benefit. The sponge construct are designed to bind its complementary miRNA(s) or their seed sequences, and hence block the binding of the miRNA to its biological targets (29–31). Here, we have designed a sponge sequence against miR-21, packaged the construct into exosomes of the transfected cells, and introduced the engineered exosomes to glioblastoma cell lines, U87-MG and C6. Our data revealed that the exosomes containing miR-21-sponge have a therapeutic potential to combat glioblastoma.

## Materials and Methods

### Plasmid Constructions

miR-21-sponge was constructed by including three miR-21 complementary sequences (HGNC: 31586) joined to each other by linkers and cloned into Tracer vector (Supplementary Table 1). Pri-miR-21 DNA was also cloned into pLentiIII vector as a control for miR-21 upregulation. The accuracy of cloning procedures was confirmed by DNA sequencing (Macrogene, South Korea).

### Cell Culture and Transfection

HEK-293T cells was obtained from the Iranian biological resource center and cultivated in DMEM-F12 media (Gibco, USA), containing 10% fetal bovine serum (FBS, Gibco, USA) and 1% penicillin-streptomycin (Bio Basic, Canada) and seeded in 12-well cell culture plates (SPL Life Science, South Korea). Stable cell line colonies expressing pri-miR-21 and miR-21-sponge, as well as their mock vectors were generated by transfecting the cells at a confluency of 70%, using lipofectamin 3000 (Invitrogen, USA). Stable cell line colonies were produced via antibiotic selection of 4 μg/ml Puromycin (Sigma, Germany) and 2 μg/ml Zeocin (Invitrogen, USA), then the antibiotic concentrations were reduced slowly.

### RNA Extraction and RT-PCR

Total RNA was extracted using Trizol or Trizol LS (for cell media and exosomes) reagents (Invitrogen, USA). After cDNA synthesis (Takara, Japan), real-time PCR analysis was performed to quantify miR-21 expression level via stem-loop method [(32); Supplementary Table 1], using SYBR Green reagent (Bio Fact, South Korea). The expression levels of miR-21 targets, PDCD4 (HGNC: 8763) and RECK (HGNC: 11345), and also miR-21-sponge production in transfected cells were quantified using specific primers. 5S rRNA (HGNC: 1380) was used as an internal control for miR-21 data analysis and GAPDH (HGNC: 4141) was also employed as the internal control for PDCD4 and RECK expression analysis (Supplementary Table 1).

### Co-culture Tests

To confirm miR-21 and miR-21-sponge effects on target cells, HEK-293T stably transfected cells were seeded on 6-well plates. After 24 h, U87-MG cells (obtained from Iranian biological resource center), as target cells, were seeded on inserts with 0.4 μm pores (SPL Life Science, South Korea). Twenty-four and forty-eight hours later, total RNAs of HEK-293T and U87-MG cells were extracted for gene expression analysis.

### Exosome Purification and Characterization

Stable cell lines were cultured in a T75 flask in media supplemented with exosome-free FBS [exosomes depleted by centrifugation at 100,000 g for 2.5 h; (33)]. Conditioned media were collected every 2–3 days, and exosomes were extracted by several steps of centrifugation and then solved in 100 μl of sterile PBS (300 g/10 min, 2,000 g/10 min, 10,000 g/30 min 20,000 g/60 min, 100,000 g/70 min). The size of the exosomes were then calculated by DLS analysis (with 10 min sonication) and Bradford assay was done to determine their concentrations. Scanning electron microscopy (SEM) was performed using a KYKY-EM3200 instrument. Western blotting was carried out using two specific antibodies against exosomes' membrane, anti-CD81, and anti-CD63 (Bioscience, SBI, USA). Exosomes were lysed with RIPA buffer (Santa Cruz, USA), and protein concentrations was quantified using the Bradford assay. Briefly, 20 μg of total protein was separated via the SDS-PAGE and blotted onto a PVDF membrane (Bioscience, SBI, USA). After blocking with 5% dry milk in Tris buffer saline plus 0.05% Tween (TBS-T), samples were incubated with anti-CD63 or anti-CD81 antibodies, for overnight at 4°C. Then, the blot was incubated with the goat anti-rabbit HRP-conjugated secondary antibody (Bioscience, SBI, USA) for 1 h at room temperature. Finally, visualization of the target proteins was done by the ECL kit (Santa Cruz, USA).

### Exosomes Uptake

To confirm uptake of the collected exosomes into the U87-MG target cells, exosomes pellets were stained with PKH-26 (Sigma, Germany), as instructed by the manufacturer's protocol. After 12–14 h of incubation, the treated cells were fixed and stained with DAPI.

### Exosomes Effect on U87-MG and C6 Cell Lines

U87-MG cells were seeded onto 24-well plate in RPMI media (Gibco, USA) supplemented with 10% FBS and 1% Penicillin/streptomycin antibiotics. Then, extracted exosomes were added to the cells at 50 μg/ml concentration, and after 24 and 48 h the expression levels of miR-21 and miR target genes were quantified. C6 cells (rat glioma cell line, obtained from Iranian biological resource center) were seeded onto a 24-well plate in Ham's F12 media (Biosera, France), supplemented with 10% FBS and 1% Penicillin/streptomycin antibiotics. Then, the effects of extracted exosomes on examined cells were analyzed, as described for U87-MG cells.

### MTT Assay

After seeding U87-MG cells on a 96-well plate, the extracted exosomes were added to the cells with a concentration of 50 μg per well. After 24 and 48 h, 50 mg/ml of MTT (10% of total volume) was added, and after 4 h of incubation, crystals were dissolved in DMSO and cell viability was evaluated in 570 nm. Three independent replicates were used for each experiment.

### Flow Cytometry Assay

To determine the rate of apoptosis and necrosis, we employed flow cytometry and Annexin V staining method. After seeding U87-MG cells on a 24-well plate, extracted exosomes were added to the cells at a concentration of 50 μg per well. After 24/48 h, the cells were washed out with PBS and stained with Annexin V-FITC as well as propidium Iodine solutions, before being analyzed by flow cytometry. Two independent replicates were used for each experiment.

### *In vivo* Experiments

To examine a potential therapeutic effect of engineered exosomes *in vivo*, we generated a glioblastoma xenograft rat model by stereotaxically injecting 1 million C6 cells in 10 μl of PBS at Caudate Putamen striatum (CPu, 2 mm up and right from bregma, in 4 mm depth) of Wistar male rats (250–300 g). After 11 days, the tumor production was verified by Magnetic Resonance Imaging (MRI) with T2 method. The size of the tumors were then measured by FSL (FMRIB Software Library), with a threshold-determining and manually voxel counting. To analyze the exosome effects, we stereotaxically injected 15–20 μg of extracted exosomes at the same position of rat brains and took MRI pictures again after 1 week.

### Statistical Analysis

All data repeated at least 3 times and were analyzed with ΔΔCT method by GraphPad Prism 6 software. The statistically significant changes tested with the ordinary ANOVA test. All histograms were presented as mean ±SD, and differences were considered as significant when the *P* < 0.05.

## Results

### An Engineered miR-21-Sponge Construct Bind and Inhibited miR-21 Actions

**Figure 1:**
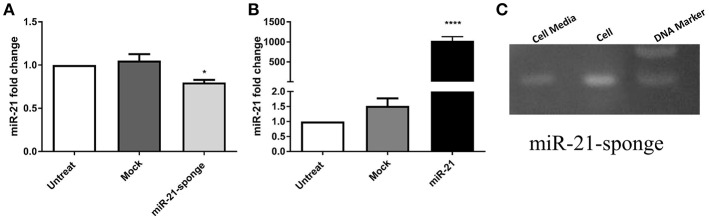
The expression level of miR-21 in HEK-293T stable cell lines exogenously expressing pri-miR-21 or miR-21-sponge. **(A)** A decline in miR-21 level (*P* < 0.05) in the cells stably expressing miR-21-sponge construct, in comparison to the untreated or stably expressing the mock-Tracer vector HEK-293T cells. **(B)** A dramatic upregulation of miR-21 (*P* < 0.0001) in HEK-293T stable cells overexpressing pri-miR-21, in comparison to the untreated or HEK-293T cells stably expressing a mock-pLentiIII vector. **(C)** An agarose gel electrophoresis showing the presence of the miR-21-sponge (94 bp) in the cell lysates and cell media of miR-21-sponge expressing HEK-293T cells. ^*^P < 0.05; ^****^P < 0.0001, which is represented by some statistical software like Graph Pad.

To block the action of miR-21, we designed a DNA construct containing three miR-21 complementary sequences, and cloned it into the Tracer vector. We also cloned a DNA segment containing pri-miR-21 sequence, and cloned it into the pLentiIII vector. In stably transfected HEK-293T cells with the recombinant vectors, the expression level of miR-21 was measured via real-time PCR (Supplementary Figure 1). According to our data, the overexpressed miR-21-sponge has the potential to reduce miR-21 level in transfected cells (*P* < 0.05, Figure 1A), in comparison to the cells stably transfected with an empty (mock) tracer vector and also untransfected HEK-293T cells. In stable cells overexpressing pri-miR-21, the expression level of miR-21 was elevated as much as 1,000 times, in comparison to the untransfected HEK-293T cells (*P* < 0.0001, Figure 1B).

Specific primers were also employed to confirm the expression level of miR-21-sponge construct in stably transfected HEK-293T cell line, as well as in conditioned media collected from the cells (Figure 1C).

### Altered miR-21 Level in U87-MG Cells Co-cultured With Pri-miR-21 or miR-21-Sponge Expressing HEK-293T Cells

**Figure 2:**
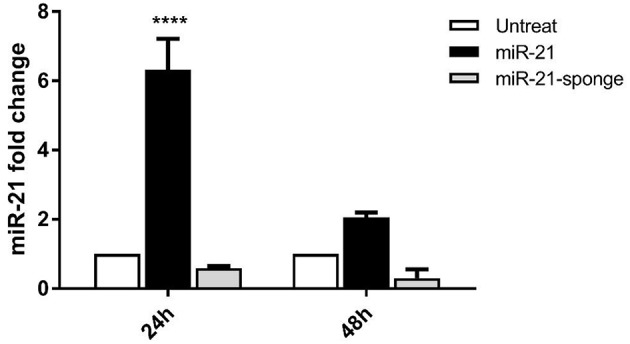
miR-21 expression level in U87-MG cells, after 24 and 48 h of co-culture with HEK-293T cells stably expressing pri-miR-21 or miR-21-sponge cells. The results revealed a potential transfer of miR-21 and miR-21-sponge from HEK-293T to U87-MG cells via conditioned media. ^****^P < 0.0001, which is represented by some statistical software like Graph Pad.

The glioblastoma cell line, U87-MG, is used to examine a potential effect of secreted miR-21 and miR-21-sponge in a co-culture system with the HEK-293T stably transfected cells. After 24 and 48 h of conditioned media contact between U87-MG and pri-miR-21 or miR-21-sponge expressing HEK-293T cells, miR-21 expression level was quantified with a real-time RT-PCR approach. Our data revealed that secreted miR-21-sponge can be transferred from the producing cells to the U87-MG cells and reduce the level of miR-21 in the target cells (Figure 2). Similarly, the secreted miR-21 had a similar potential in elevating the level of miR-21 in co-cultured U87-MG cells. Although the secreted miR-21 had a significant effect in U87-MG's miR-21 level after 24 h of co-culture, however, the effect of miR-21-sponge was more evident after 48 h of treatment.

### The Secreted miR-21 and miR-21-Sponge Was Packaged Inside Exosomes

To confirm a potential exosomes packaging of secretory miR-21 and miR-21-sponge of the stably transfected HEK293T cells, the exosomes were extracted via ultracentrifugation of the serum depleted conditioned cell media. To confirm the identity of the exosomes, the size of the extracellular vesicles were determined with DLS, which demonstrated a single peak with 66.65 nm diameter for the vesicles (Supplementary Figure 2A). Moreover, Western blot assay confirmed the presence of the specific exosomes markers on the surface of the extracted exosomes (Supplementary Figure 2B). Finally, SEM showed a <100 nm diameters of the extracted vesicles, corresponding to the size of the typical exosomes (Supplementary Figure 2C). Next, we confirmed the integration of the PKH-26 labeled exosomes to the cell membranes of the target cells, 12 h after incubation (Supplementary Figure 3).

### miR-21 and miR-21-Sponge Packaged Within Engineered Exosomes

**Figure 3:**
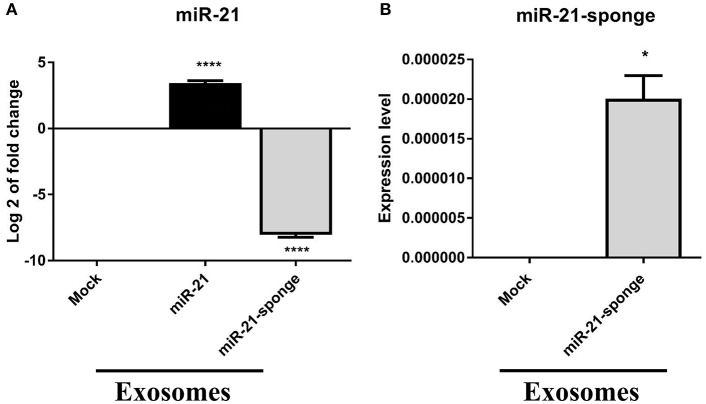
miR-21 levels in extracted exosomes from miR-21 and miR-21-sponge expressing HEK-293T cells. **(A)** Log2 of miR-21 levels in extracted exosomes from stable HEK-293T cells exogenously expressing miR-21, in comparison with exosomes extracted from HEK-293T cells expressing mock-pLentiIII, revealed a significant elevation in miR-21 level (*P* < 0.0001). Similarly, miR-21 level demonstrated a significant decline in extracted exosomes obtained from miR-21-sponge expressing HEK-293T cells, compared to the exosomes extracted from HEK-293T cells stably expressing mock-Tracer vector (*P* < 0.0001). **(B)** The packaging of miR-21-sponge in exosomes extracted from miR-21-sponge expressing HEK-293T stable cell line, note that its expression was undetermined in HEK-293T expressing mock-Tracer. ^*^P < 0.05; ^****^P < 0.0001, which is represented by some statistical software like Graph Pad.

miR-21 level in exosomes extracted from pri-miR-21 expressing HEK-293T stable cell line represented an approximately 10 folds elevation, compared to those of mock-pLentiIII expressing HEK-293T stable cell line (*P* < 0.0001). A decline in the level of endogenous miR-21 in exosomes enriched from the conditioned media from miR-21-sponge expressing HEK-293T stable cell line was also observed, in comparison with those of mock-Tracer expressing HEK-293T stable cell line (*P* < 0.0001, Figure 3A). Accordingly, a packaging of miR-21-sponge construct was demonstrated in exosomes enriched from conditioned media of the miR-21-sponge expressing cells (Figure 3B).

### The Effects of the Engineered Exosomes Treatment on U87-MG Cells

**Figure 4:**
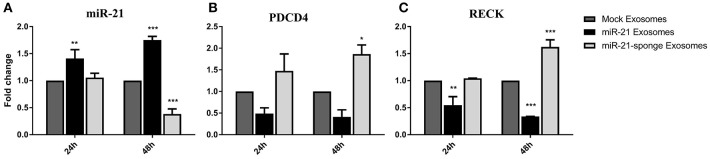
Altered expression of miR-21 and its target genes in U87-MG cells exposed to engineered exosomes. **(A)** A significant elevation of miR-21 level in U87-MG cells exposed for 24 or 48 h to the exosomes obtained from pri-miR-21 expressing HEK-293T stable cells (miR-21 enriched exosomes), along with a downregulation of miR-21 in the cells treated with exosomes obtained from the miR-21-sponge expressing HEK-293T stable cell line (miR-21-sponge containing exosomes). The expression levels of miR-21 target genes PDCD4 **(B)** and RECK **(C)** revealed their expected downregulations in the cells exposed to miR-21 enriched exosomes and their upregulations in the cells exposed to the miR-21-sponge containing exosomes, at 24 and 48 h after the treatments. Exosomes obtained from mock-vectors transfected HEK-293T stable cell lines were used as controls to normalized the levels of gene expressions. ^*^P < 0.05, ^**^P < 0.01, ^***^P < 0.001, which is represented by some statistical software like Graph Pad.

To evaluate any potential therapeutic effects of engineered exosomes, we quantified the levels of miR-21 and its well-known targets on U87-MG cells exposed to the exosomes enriched for either miR-21 or miR-21-sponge. Our results revealed that the level of miR-21 was significantly altered by transferred miR-21 and miR-21-sponge containing exosomes to the U87-MG target cells after 24 and 48 h of treatment. A significant increase in the level of miR-21 was evident after treating the U87-MG cells with miR-21 enriched exosomes at 24 h (*P* < 0.01) and 48 h (*P* < 0.001) of treatment. In contrast, a decline of endogenous miR-21 in the cells exposed to the miR-21-sponge enriched exosomes was statistically significant only at 48 h post-treatment (*P* < 0.001; Figure 4A).

To explore the functional effects of the engineered exosomes on target cells, their effects were also examined on two important miR-21 target genes, *PDCD4* and *RECK*. As expected, an elevation of miR-21 in the cells exposed to the miR-21 enriched exosomes followed by a significant down-regulation of *PDCD4* and *RECK* in U87-MG cells. Similarly, a decline in the level of miR-21 in the U87-MG cells treated with miR-21-sponge containing exosomes followed with an upregulation of *PDCD4* and *RECK*. Again, the effect was statistically significant only at 48 h post-treatment (Figures 4B,C). Exosomes extracted from the mock-vectors expressing cells, were used as controls to normalize the level of gene expression in all above mentioned experiments.

### miR-21-Sponge Enriched Exosomes Decreased the Cell Viability Rate of U87-MG Cells

**Figure 5:**
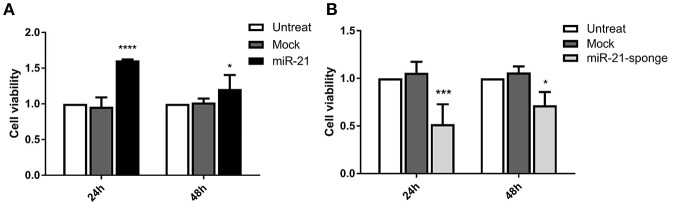
Cell viability assay in the U87-MG cells exposed to the engineered exosomes. **(A)** The MTT assay (MTT absorbance at 570 nm) demonstrated a significant increase in the cell viability of U87-MG cells treated with miR-21 enriched exosomes (*P* < 0.0001 at 24 h, and *P* < 0.05 at 48 h). **(B)** MTT absorbance in U87-MG cells revealed a significant decreased in cell viability, in the cells exposed to the miR-21-sponge containing exosomes (*P* < 0.001 at 24 h, and *P* < 0.05 at 48 h). The cells exposed to the mock-vectors containing exosomes were used as controls. ^*^P < 0.05; ^***^P < 0.001; ^****^P < 0.0001, which is represented by some statistical software like Graph Pad.

Our MTT assay data revealed that the exposure of U87-MG cells with engineered exosomes enriched for miR-21 or miR-21-sponge can affect cell viability of the treated cells. MTT absorbance alteration was more significant at 24 h post-incubation for both miR-21 (*P* < 0.0001) and miR-21-sponge (*P* < 0.001) enriched exosomes. After 48 h of treatment, MTT absorbance alteration was still significant (*P* < 0.05), however, the level of alterations was lower than that of the 24 h time point (Figure 5).

### miR-21-Sponge Enriched Exosomes Increased the Cell Death Rate in Treated U87-MG Cells

**Figure 6:**
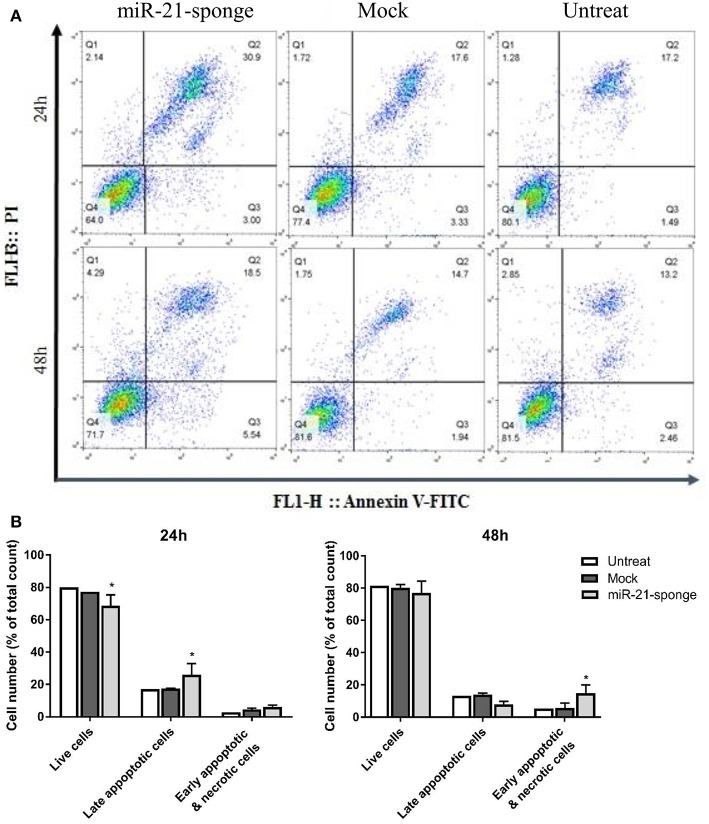
The apoptosis rate in U87-MG cells exposed to the engineered exosomes. **(A)** A typical quadrant analysis of Annexin V-FITC/PI flow cytometry of U87-MG cells after 24 and 48 h of treatment with miR-21-sponge, compared to the exosomes extracted from the mock-Tracer vector expressing and untransfected HEK293T cells. The proportion (%) of cell numbers is shown for each quadrant. The proportion of viable cells was shown in Q3 quadrant (FITC−/PI−), early apoptotic cells shown in Q4 quadrant (FITC+/PI−), late apoptotic cells shown in Q2 quadrant (FITC+/PI+), and necrotic cells shown in Q1 quadrant (FITC−/PI+). **(B)** An increased late apoptotic cell numbers and an decreased alive cell numbers in U87-MG cells treated with miR-21-sponge containing exosomes was evident after 24 h (*P* < 0.05). At 48 h, there was no significant difference in the late apoptotic and alive cell numbers, however, a significant increase in the early apoptotic and necrotic cell numbers was observed (*P* < 0.05). ^*^P < 0.05, which is represented by some statistical software like Graph Pad.

In a continuation of the MTT experiment, we employed Annexin V-FITC and PI staining methods to investigate the rate of apoptosis in the U87-MG cells treated with miR-21-sponge containing exosomes. Our data revealed an elevated cell death rate (most prominently after 24 h of treatment) in the cells exposed to the exosomes enriched for miR-21-sponge, compared to the exosomes extracted from the mock-Tracer vector expressing and untreated cells. At 24 h after treatment, miR-21-sponge containing exosomes caused an increase (up to 30.9%, compared to 17% of untreated cells) in the rate of late apoptosis (Q2) and a decrease in the percentage of the alive cells (Q4), from 80% on untreated samples to 64% in miR-21-sponge treated samples (Figure 6). At 48 h post-treatment, less effects on Q2 and Q4 were seen, in comparison with the 24 h treatment group. However, an increase in the percentage of the cells in early apoptosis (Q3) and necrotic cells (Q1) was observed (respectively, 5.54 and 4.29% of total cell count; Figure 6). Altogether, our data revealed that miR-21-sponge containing exosomes have the potential to cause apoptosis and reduce cell viability in U87-MG target cells, through miR-21 inhibition.

### The Therapeutic Effects of Exosomes Containing miR-21-Sponge on C6 Cells

**Figure 7:**
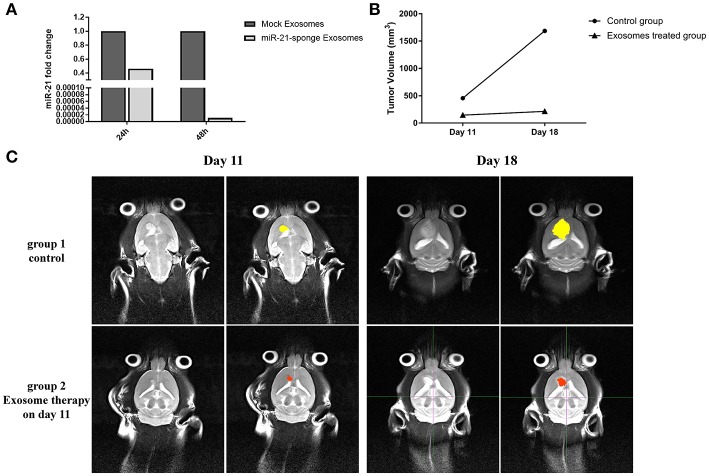
*In vivo* analysis results: **(A)** Downregulation of miR-21 in the C6 cells treated with exosomes enriched with miR-21-sponge at 24 and 48 h in comparison with cells treated with exosomes obtained from mock-vectors transfected HEK-293T stable cell lines observed. **(B)** Tumor volume graph on day 11 and 18 after stereotaxically injecting 1 million C6 cells at CPu position on rat brains. The exosomes treated group received about 15 μg exosomes enriched with miR-21-sponge on day 11 and control groups had no treatment. **(C)** MRI pictures from two rats on day 11 and 18 from. The exosomes treated rat's tumor had growth inhibition on day 18 in comparison with control rat.

We quantified the level of miR-21 on C6 cells exposed to the exosomes enriched for miR-21-sponge. Our data revealed that the level of miR-21 in C6 cells was significantly altered by exposure to the miR-21-sponge containing exosomes after 24 and 48 h of treatment, similar to what we observed for U87-MG cells. A decrease of endogenous miR-21 in the cells exposed to the miR-21-sponge containing exosomes was observed, that was more visible at 48 h post-treatment (Figure 7A).

### Therapeutic Potential of Administrating miR-21-Sponge Exosomes on a Rat Model of Glioblastoma

**Figure 8:**
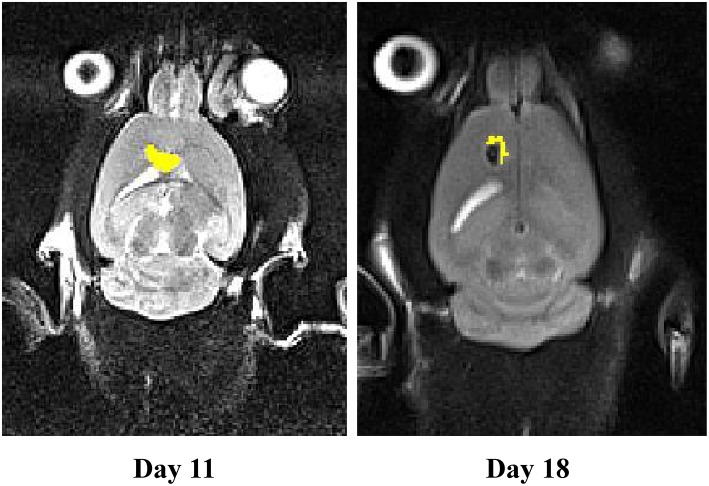
The MRI results on a rat model of glioblastoma treated with fresh exosomes containing miR-21-sponge. Results from the third repeat treated with freshly prepared exosomes containing miR-21-sponge demonstrated good effects not only in inhibiting tumor growth, but also in eradicating tumor mass. Note the black area in the middle of the treated tumor on day 18.

We measured tumor volume (mm^3^) at day 11 of stereotaxically injecting 1 million C6 cells in rat brain. One group of rat xenograft model received 15–20 μg of miR-21-sponge containing exosomes and the tumor's volume were measured again at day 18. The latter result revealed that administrating miR-21-sponge packed exosomes in rat brain could repress tumor growth rate in comparison with untreated as well as unmodified exosome treated animals (Figures 7B,C). In all three repeats, we observed a significant retardation of tumor growth in the group of miR-21 sponge containing exosomes administration, in comparison to untreated and unmodified exosome administering groups. Interestingly, the best results were achieved in glioblastoma models treated with freshly prepared modified exosomes (without freeze thawing). In this experimental repeat, the volume of the tumor decreased by 50% between day 11 and 18th. Also evidence of tumor necrosis occurrence was evident in some parts of the tumor (a dark appearance in the middle of tumor volume; Figure 8).

## Discussion

Glioblastoma multiform (GBM) is the most aggressive form of brain tumors, glioma. It is highly infiltrative and invasive, creating difficulties which need to be overcame when searching for a cure. Despite the current standard of care that combines surgery, radiotherapy and chemotherapy, GBM patients are nonetheless confronted with frequent recurrence, and hence their life expectancy is very short (1, 2, 34). Therefore, introducing novel techniques and potential molecular cures for clinical trials have a higher justification for GBM.

The extracellular vesicles, exosomes, are currently considered an important tool for extracellular communications and clinical process (35–37). Similar to other cancer cell types, GBM cells employ exosomes for tumor cells/microenvironment communications and to facilitate its proliferation, invasion, and metastatic behaviors (8, 9). One of the major challenges in exosome-based therapeutics is low productivity of exosomes. Thus, effective large-scale exosome production methods are required. Watson et al. (38) showed that yield of exosomes can be increased by 5–10 fold using a hollow fiber bioreactor. However, whether yield of exosomes was actually increased by use of the bioreactor is not clear, since the obtained sample contained larger vesicles (200–800 nm in diameter). Other topics about exosomes extraction for therapeutic approach remained under investigation, such as best isolation methods, collection of high-quality and uniform exosomes, optimization of storage conditions, improvement of therapeutic potential of exosomes, and delivery of exosomes (38–41). Despite all exosomes challenges for their optimal use, they are expected to become effective therapeutic reagents for various diseases and provide an enormous promise and a fresh therapeutic area for delivery of different synthetic and biological molecules in cellular therapy.

Numerous miRNAs with oncogenic and tumor-suppressive properties have been identified in GBM (42). As we have currently reported (43), the upregulation of miR-21 in esophageal tumors was mainly confined to the fibroblasts within the vicinity of the tumor cells. Moreover, the co-culture experiment confirmed that miR-21 works as a micro-environmental signaling molecule to promote invasion behavior of tumor cells (43). The latter findings suggest a logical base for blocking the effect of miR-21 in tumor microenvironment as a possible therapeutic approach to combat tumor progression and invasion.

microRNA's presence in exosomes have been already reported in different cells and cancer types (44–50). Moreover, miR-21 has been demonstrated to be packaged within exosomes and released from different cancer types (51–54). In addition, miR-21 silencing has demonstrated effective results both *in vitro* and *in vivo* (24–28). Suppressing miR-21 also enhanced the therapeutic effects of antiangiogenic drug, sunitinib, in glioblastoma (55).

Using decoy or sponge-like constructs for miRNA inhibition have been already shown before with good effect in binding to its complementary miRNA(s) or their seed sequences and blocking their effects (29–31, 56, 57).

Here, we have constructed a miR-21-sponge and packaged it into secretory exosomes. Introducing the engineered exosomes to U87-MG and C6 cells demonstrated a potential therapeutic suitability of the miR-21-sponge in combat GBM and probably other types of cancers. The exosomes packed with miR-21-sponge could easily applied to cancer cells without any need for transfecting reagents.

Our data on the effects of miR-21 enriched exosomes is in agreement with the previous reports demonstrating that overexpressed miR-21 in cell lines can increase the proliferation rate and malignant behavior of the cells (17–23). On the other hand, as expected, exosomes filled with miR-21-sponge downregulated miR-21 in U87-MG and C6 cells and upregulated *PDCD4* and *RECK* in U87-MG target cells. The latter results approved the suitability of the miR-21-sponge packaged exosomes to combat GBM, and probably other types of cancers.

To confirm our results, we also employed C6 cell line for generating xenograft rat models, because of a close gene expression pattern to human brain tumors and they are also widely used in neuro-oncological studies (58–60). Our *in vivo* results on the rat model of glioblastoma, demonstrated the effectiveness of the manipulated exosomes in repressing tumor growth and inducing tumor retardation. The latter result was more prominent, when we employed freshly prepared engineered exosomes. Exosomes stability in different PH, temperature and other conditions has been already studied and reported. Moreover, exosomes modifications under a different situation of maintenance and with a different cycle of freeze thawing has been already reported (61, 62). We can conclude that freshly prepared exosomes have a better suitability for *in vivo* applications.

The existence of the blood-brain-barrier introduces a unique challenge for applying cell-based gene therapy of brain tumors (63, 64). Importantly, the engineered exosomes could cross the multiple layers of the blood-brain-barrier (BBB) and target cancer cells within the brain tissue (40, 65–68). It is safe to suggest that the engineered exosomes have the potential to successfully cross the BBB and transfer their contents within the brain.

## Data Availability

The raw data supporting the conclusions of this manuscript will be made available by the authors, without undue reservation, to any qualified researcher.

## Ethics Statement

This research was checked on ethics committee of Tarbiat Modares University of Iran and confirmed with below ethic code: IR.MODARES.REC.1398.050.

## Author Contributions

HM designed and performed experiments, analyzed data, and wrote the paper. YJ helped in performing experiment. MN and JM-Z provided consultations and supervised the project. SM supervised the research, designed the experiments, analyzed data, and wrote the paper.

### Conflict of Interest Statement

The authors declare that the research was conducted in the absence of any commercial or financial relationships that could be construed as a potential conflict of interest.

## Supplementary Material

Supplementary Figure 1 Transfection of pri-miR-21 and miR-21-sponge containing vectors into HEK-293T cells. GFP used as a reporter to visualize transfected cells at 48 h **(A)** and 15 days post-transfection **(B)**, as well as in stable cells **(C)**.

Supplementary Figure 2 Characterizing the purity and nature of the extracted exosomes. **(A)** Our exosome's size with DLS represented good quality and distribution with a unique size peak under 100 nm (66.65 nm). **(B)** Western bloting with special antibodies, anti CD63, and anti CD81, revealed accurate staining of the exosomes membrane special markers and correct negative answer of markers in cell lysate. **(C)** SEM photos with 4 × 10^4^ and 2 × 10^4^ indicated particles size between 30 and 170 nm, mostly under 100 nm.

Supplementary Figure 3 Staining with PKH-26 confirmed exosomes entrance to target cells. Exosomes membrane stained with PKH-26 (showed in red) and Fixation and target cell nucleus, U87-MG, staining with DAPI (showed in blue) was done after 12 h, confirmed exosomes entrance to U87-MG target cells.

Supplementary Table 1 Primers and other used sequences.
