# Supplementary figures and images for: Potential Therapeutic Effects of Exosomes Packed With a miR-21-Sponge Construct in a Rat Model of Glioblastoma
Source: Front Oncol. 2019 Aug 20;9:782. doi: 10.3389/fonc.2019.00782 (PMC6710330; doi:10.3389/fonc.2019.00782)

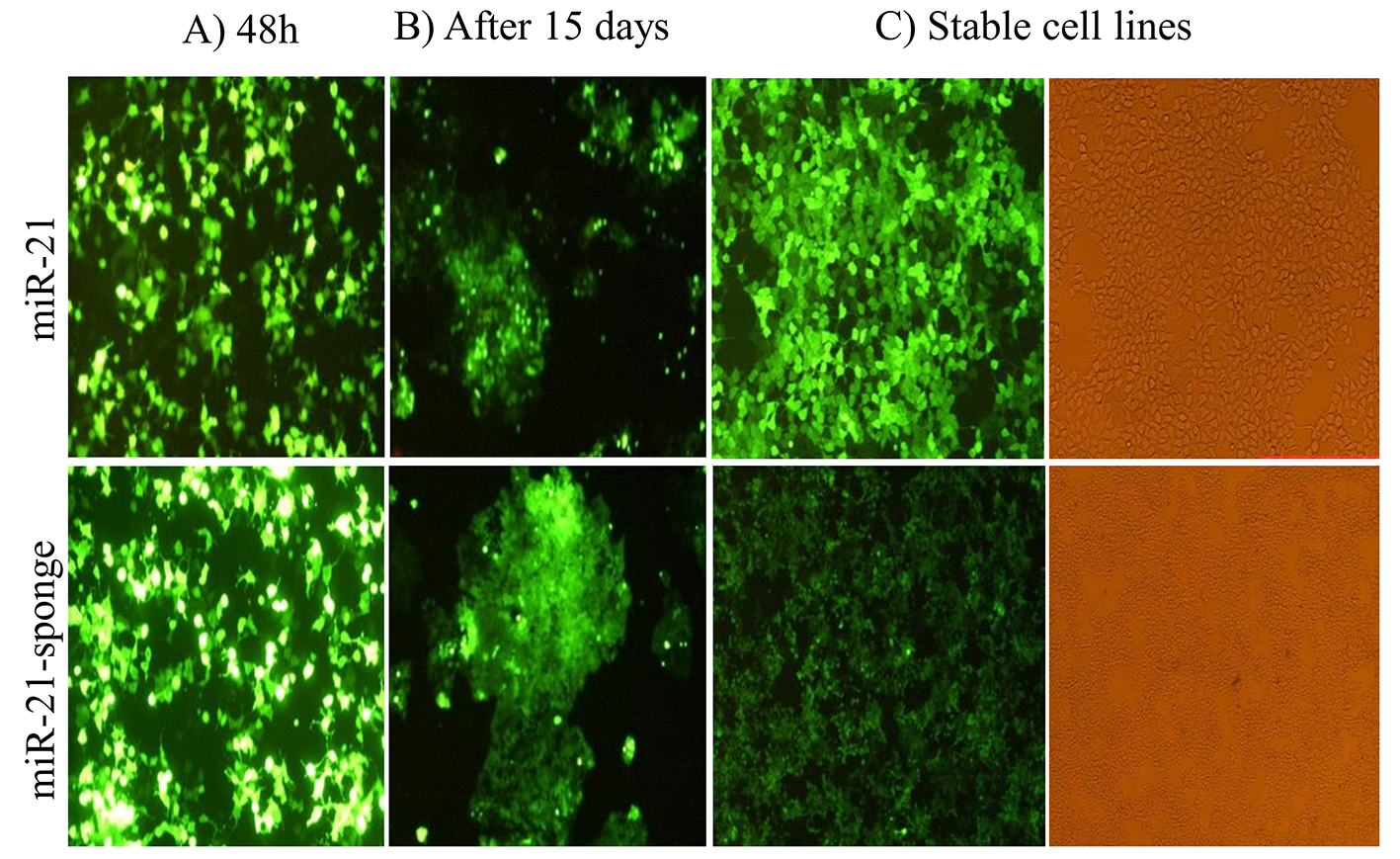

Supplement: Supplementary Figure 1 — Transfection of pri-miR-21 and miR-21-sponge containing vectors into HEK-293T cells. GFP used as a reporter to visualize transfected cells at 48 h (A) and 15 days post-transfection (B), as well as in stable cells (C). [file Image_1.TIF]

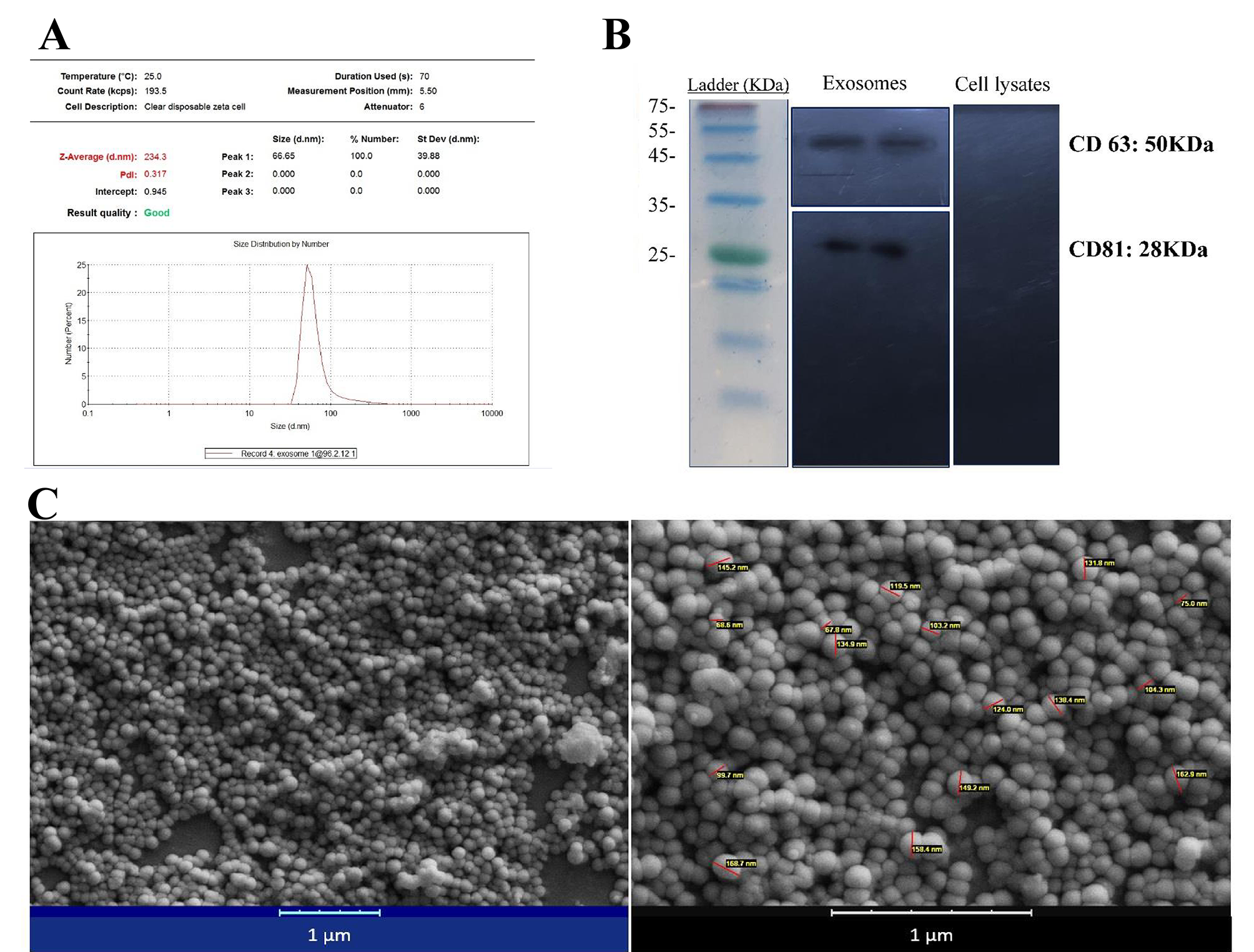

Supplement: Supplementary Figure 2 — Characterizing the purity and nature of the extracted exosomes. (A) Our exosome's size with DLS represented good quality and distribution with a unique size peak under 100 nm (66.65 nm). (B) Western bloting with special antibodies, anti CD63, and anti CD81, revealed accurate staining of the exosomes membrane special markers and correct negative answer of markers in cell lysate. (C) SEM photos with 4 × 10^4^ and 2 × 10^4^ indicated particles size between 30 and 170 nm, mostly under 100 nm. [file Image_2.TIF]

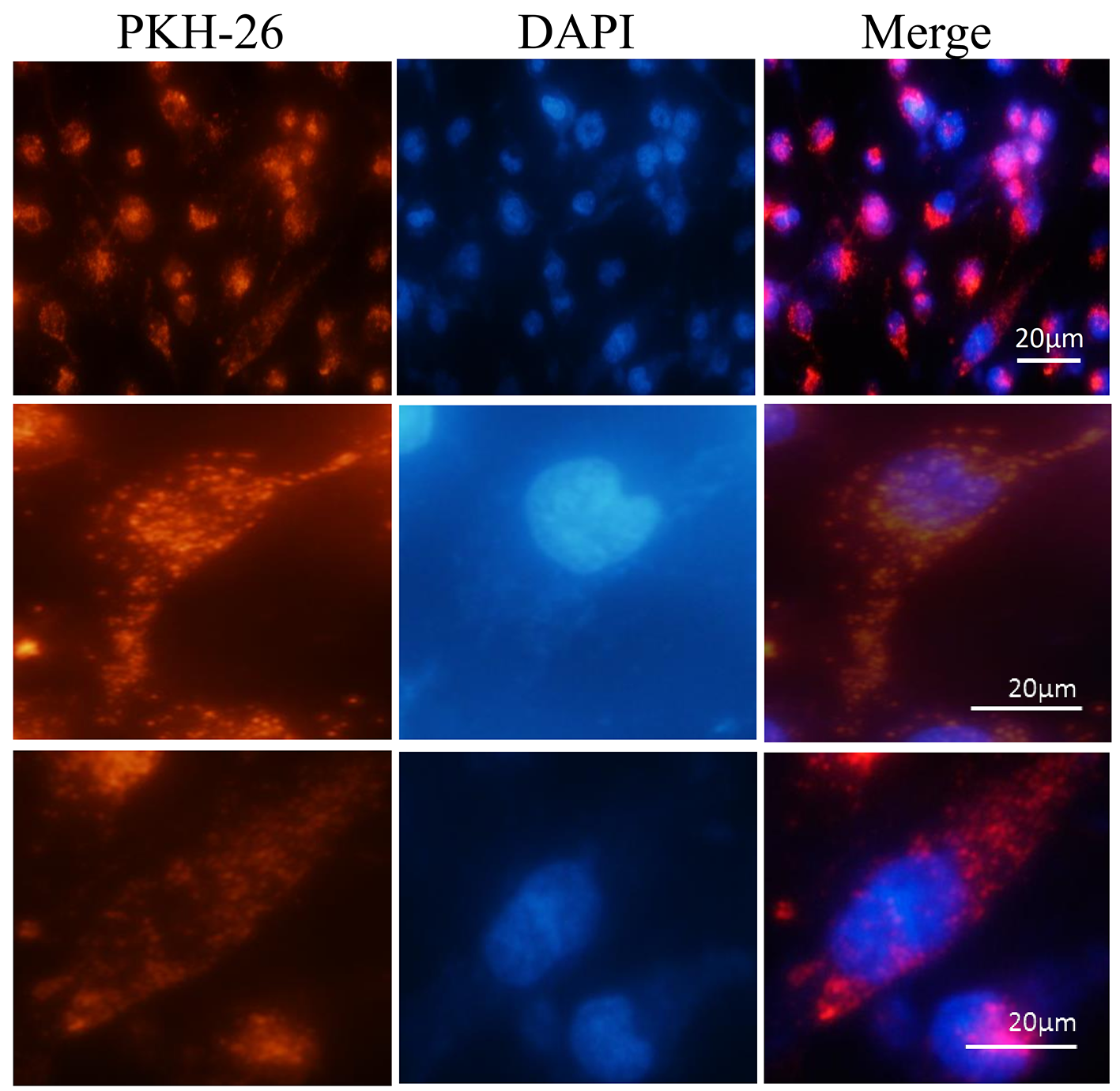

Supplement: Supplementary Figure 3 — Staining with PKH-26 confirmed exosomes entrance to target cells. Exosomes membrane stained with PKH-26 (showed in red) and Fixation and target cell nucleus, U87-MG, staining with DAPI (showed in blue) was done after 12 h, confirmed exosomes entrance to U87-MG target cells. [file Image_3.TIF]

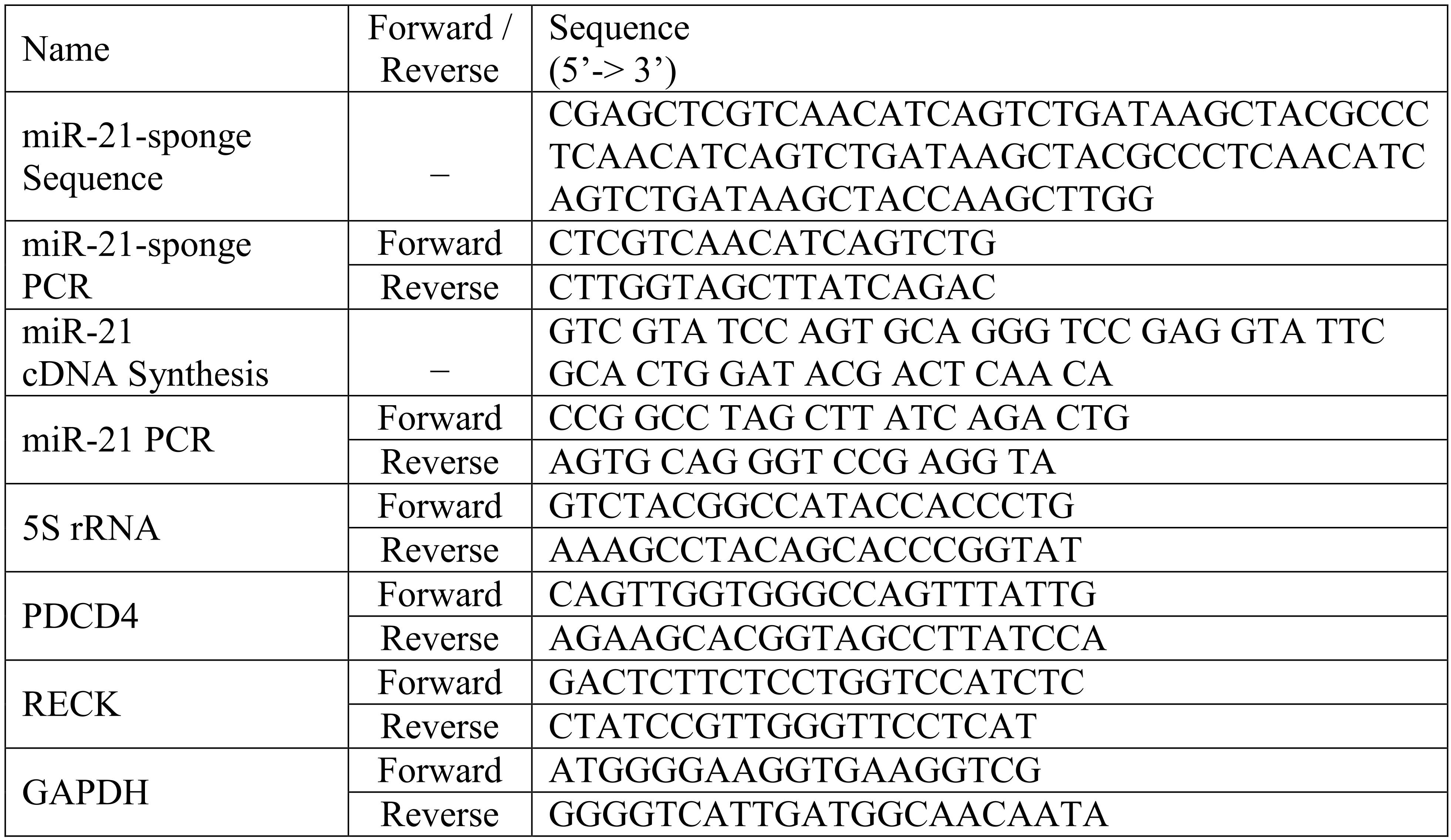

Supplement: Supplementary Table 1 — Primers and other used sequences. [file Image_4.TIF]
